# Baseline characteristics and the factors influencing successful smoking cessation: A comparison between a WeChat smoking cessation mini-program and an offline smoking cessation clinic

**DOI:** 10.18332/tid/174491

**Published:** 2023-11-22

**Authors:** Lei Zhu, Yanfang Qiu, Rui Zhong, Jianghua Xie, Yina Hu, Xinhua Yu, Xiaochang Chang, Wei Wang, Lemeng Zhang, Ouying Chen, Hui Cao, Haidong Zhu, Yanhui Zou

**Affiliations:** 1 Hunan Cancer Hospital, The Affiliated Cancer Hospital of Xiangya School of Medicine, Central South University, Changsha, China; 2 School of Nursing, Hunan University of Chinese Medicine, Changsha, China; 3 Xiangya Hospital, Central South University, Changsha, China; 4 School of Nursing and Health Management, Wuhan Donghu University, Wuhan, China; 5 Hunan Yixuan Technology Co., LTD, Changsha, China

**Keywords:** smoking cessation, smoking cessation clinic, WeChat mini-program, smoking cessation rate, predictors

## Abstract

**INTRODUCTION:**

Smoking cessation (SC) clinics are a professional SC services in China. However, studies comparing the characteristics and SC rates of smoking populations in SC clinics with those using mobile SC programs are limited. We compared smokers’ characteristics, 3-month SC rates, and the factors influencing 3-month SC success, between a large hospital SC clinic and a WeChat SC mini-program.

**METHODS:**

Between January and November 2021, 384 participants voluntarily enrolled in either the hospital SC clinic (Group A: n=243) or the WeChat SC mini-program (Group B: n=141). Both groups underwent a 3-month SC intervention, and their SC status was monitored at 24 hours, 1 week, 1 month, and 3 months after quitting. SC rate was defined as the self-reported rate of continuous SC.

**RESULTS:**

The 3-month SC rate was higher in Group A (42.4%) than in Group B (24.8%). Participants with middle school education had a lower likelihood of SC success than those with primary school or lower (p=0.014). Employees in the enterprise/business/services industries were more likely to have SC success than farmers (p=0.013). Participants with SC difficulty scores of 0–60 were more successful than those with scores >60 (p=0.001, p=0.000, respectively). Participants who quit smoking due to their illness, or other reasons, had a higher likelihood of SC success than those who quit due to concerns about their own and their family's health (p=0.006, p=0.098, respectively). While the likelihood of SC success was lower in those who quit because of the influence of their environment than in those who quit due to concerns about their own and their family's health (p=0.057).

**CONCLUSIONS:**

Both SC clinics and WeChat SC mini-programs achieved satisfactory SC rates. The high accessibility of mobile SC platforms, which save time spent on transportation and medical visits, renders them worth promoting and publicizing as additional SC options for smokers, particularly young smokers.

## INTRODUCTION

The number of adult smokers in China (approximately 308 million) ranks first in the world, and the adult smoking rate in China (26.6%) is higher than that of the global smoking rate of 19.2%; however, the smoking cessation (SC) rate is only 20.1%^1^. SC clinics are a common form of professional SC services in China^2,3^. The 7-day point prevalence abstinence during 3-month follow-up in SC clinics in China was 28.4%, and the sustained SC rate at the 3-month follow-up was 19.9%^4^. The SC rate among adult smokers aged ≥18 years in Gansu, China, was 16.4% (380/2311)^5^.

With advancements in mobile medical technology, mobile SC platforms, including SC mini-programs such as SmokeFree28 and Quittr, have emerged as new forms of SC treatment because of their convenience, low cost, and diverse functionality^6,7^. Ubhi et al.^6^ found that the self-reported SC rate for ≥28 days recorded by the SmokeFree28 mobile application was 18.9% and that the SC rate of SmokeFree28 users was slightly higher than that of those who did not use the application. Pallejà-Millán et al.^8^ found that the sustained SC rate for 3 months was significantly higher among participants using the Tobbstop application than in those who did not use the application (38.5% vs 13.4%).

Populations who smoke and seek SC through different methods exhibit different characteristics. The population seeking assistance from SC clinics predominantly consists of middle-aged males with lower educational levels and farmers. Zhao et al.^9^ analyzed the characteristics of smokers in 16 SC clinics in North China, and found that most smokers seeking SC assistance were middle-aged males. Similarly, another study reported that smokers who voluntarily sought SC interventions at clinics had mostly high school or lower education level, were male, farmers, with an average age of 49.22 years^10^. In contrast to older smokers, younger individuals with higher level of education were more likely to use mobile applications for SC. Fradkin et al.^11^ analyzed the demographic characteristics of 1280 registered and activated adult users of a free tobacco cessation smartphone app, and their study revealed that 57.5% (682/1186) of the app users were <45 years, and 93.7% (1145/1222) had a high school education or higher.

The effects of SC differ based on education level, occupation, and the reasons for quitting smoking. Occupation is a significant factor influencing SC outcomes, and reasons for quitting smoking also have certain effects^10^. Zeng et al.^12^ reported that individuals with lower education level showed a 51.0% reduction in the frequency of opening the SmartQuit application in comparison with those having a higher education level. Bao et al.^10^ found that 87.6% of smokers who voluntarily sought SC interventions at clinics were motivated by personal illness and concerns about their own and their family’s health. Li et al.^13^ showed that 67.5% of smokers found it difficult to quit smoking (QS), 80.3% felt it was easier to smoke with other smokers, and 17.1% experienced a relapse influenced by other smokers around them, leading to failed SC attempts. Lin et al.^4^ stated that attempts to QS under the influence of the surrounding environment were a strong independent predictor of successful SC (OR=4.14; 95% CI: 1.27–13.44). Bo et al.^14^ showed that 93.0% of smokers rated the difficulty to QS as >5 points during the 3-month follow-up.

Nevertheless, studies that have directly compared the basic characteristics and SC rates of smoking populations in SC clinics with those using mobile SC programs are limited. Comparing the characteristics and SC rates of users of online SC platforms, such as SC mini-programs, with those attending offline SC clinics would help to identify the differences and influencing factors of successful SC between these two groups of smokers, potentially facilitating the improvement and promotion of both online and offline SC services.

WeChat is a social networking platform in China that enables quick information sharing through its network. It supports multi-group chats, similar to WhatsApp. In recent years, the WeChat mini-program has emerged as a new model for delivering medical services. It has been applied in appointment scheduling, remote consultations, and other healthcare aspects, facilitating the efficient allocation of medical resources and time^15^. This has resulted in reduced patient waiting times and improved overall hospital work efficiency.

The ‘QuitAction’ WeChat mini-program (hereinafter referred to as the SC mini-program) was developed by our research team according to the China Clinical Guidelines for Smoking Cessation (2015 edition)^16^. And the ‘QuitAction’ intervention model was constructed according to the 5As (ask, advice, assess, assist, arrange) and 5Rs (relevance, risk, reward, roadblocks, repetition) methods^16^. The 5As and 5Rs methods were used as the theoretical framework for psychological counseling and support. SC professionals provide specific intervention models according to the stage of SC, and provide services such as SC counseling^17^. Smokers can chat with medical staff in real time, and view their smoking cessation results in the user’s personal center interface of the WeChat mini-program according to their needs.

Therefore, we conducted a cross-sectional study to compare the demographic and smoking characteristics and 3-month SC rates between participants in the SC clinic and the WeChat SC mini-program. We also sought to explore the factors influencing successful SC at the follow-up at 3 months.

## METHODS

### Participant recruitment

The study recruited smokers who voluntarily participated in SC clinics or the ‘QuitAction’ WeChat mini-program designed by our research team from January to November 2021. The participants were divided into two groups: the SC clinic group (Group A) and the WeChat mini-program group (Group B). Inclusion criteria were: 1) age ≥15 years, 2) smoking ≥1 cigarette/day for more than 6 months, 3) willingness to QS, and 4) Group B participants needed to be proficient in using smartphones. Individuals with severe life-threatening illnesses or cognitive impairments were excluded.

### Smoking cessation interventions

*Group A*

Participants in Group A completed a survey collecting demographic and smoking-related data under the guidance of healthcare professionals during their first visit to the clinic. They received face-to-face counseling and SC guidance. The SC clinic conducted standardized telephone follow-up of the participants at 24 hours, 1 week, 1 month, and 3 months after the initial consultation, to evaluate their SC status and provided timely counseling support based on any issues.

*Group B*

**Figure 1:**
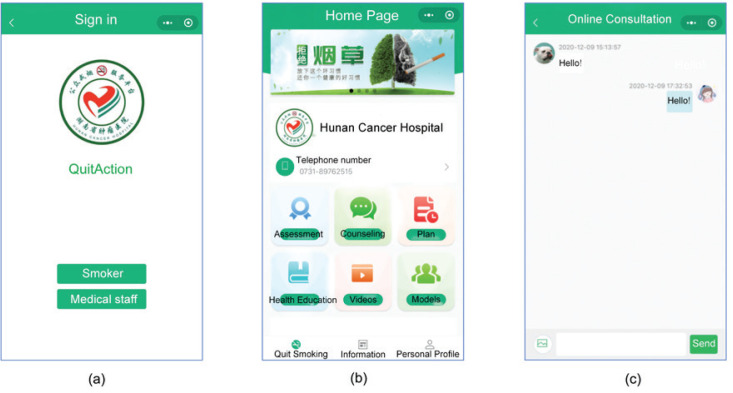
(a) Login Page, (b) Home Page, (c) Online Consultation

Participants in Group B used the ‘QuitAction’ WeChat mini-program developed by our research team for SC (this is the first time that the ‘QuitAction’ WeChat mini-program has been used for research purposes). Participants registered and logged in by scanning a QR code and then sequentially filled in demographic information, smoking-related data, and a nicotine-dependence assessment form guided by the program. The backend of the program performed the statistical analysis and provided the corresponding SC advice. The mini-program automatically reminded participants to assess their SC status at 24 hours, 1 week, 1 month, and 3 months after quitting smoking. If participants failed to complete the questionnaire within one day, the mini-program notified healthcare professionals to conduct telephone follow-up. Additionally, the mini-program included other features, such as various types of counseling, information regarding SC and SC medication use, and SC videos, to enhance participants’ awareness of tobacco hazards and provide motivation for SC (Figure 1).

### Data collection and evaluation indicators

**Figure 2:**
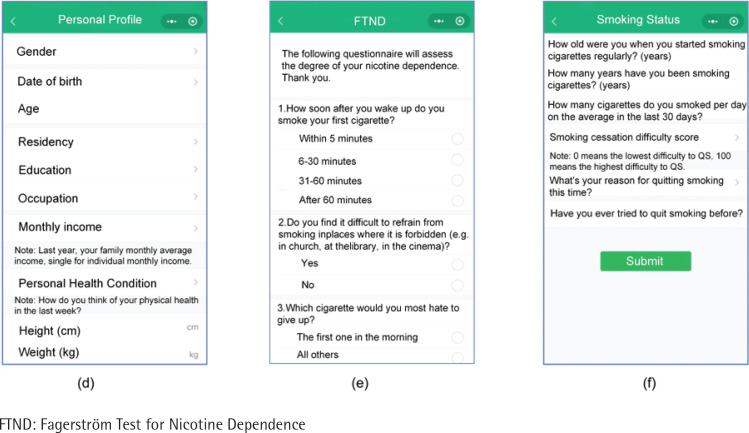
(d), (e), (f) After entering the WeChat mini program, users need to file up, personal profile, Fagerström Test for Nicotine Dependence, smoking status and other assessments. Smoking cessation professionals will give advice based on their assessment results

The assessment tools used for both groups included the ‘SC Clinic Registration Form’, ‘SC Clinic 1 Week Follow-up Questionnaire’, ‘SC Clinic 1 Month Follow-up Questionnaire’, and ‘SC Clinic 3 Month Follow-up Questionnaire’. These questionnaires were developed based on templates provided by the Tobacco Control Office of the Chinese Center for Disease Control and Prevention^16^. The collected data included information regarding participants’ gender, age, education level, occupation, height, weight, daily cigarette consumption, duration of smoking (years), previous attempts to QS, level of nicotine dependence, SC difficulty score, and reasons for quitting (Figure 2). The content of the telephone consultation was based on the follow-up questionnaire in the Chinese Clinical Guidelines for Smoking Cessation^16^, which collected general information, smoking status, SC progress, etc.

Nicotine dependence was assessed using the Fagerström test for nicotine dependence (FTND), which consists of six items with total scores ranging from 0 to 10. A score of 0 indicates no dependence, 1–3 low dependence, 4–6 moderate dependence, and 7–10 high dependence.

The SC difficulty score was determined through participants’ self-assessment of the difficulty of quitting, on a scale from 0 to 100. A higher score indicates greater difficulty in quitting.

The primary outcome of this study was the continuous SC rate of participants at three months after their initial consultation at the SC clinic or using the SC mini-program. SC success was defined as self-reported 3-month continuous SC. Participants who were lost to follow-up were defined as those with incorrect contact information, those who did not answer at least seven follow-up calls, or those who could not be reached during seven cumulative contact attempts at different periods during the study. These participants who were lost to follow-up were considered ‘non-quitters’.

### Sample size calculation

The sample size formula used in this study is as follows:


$${\text{n}}_{\text{1}}={\text{n}}_{\text{2}}=1641.4\times {\left[\frac{({\text{u}}_{\text{α}}+{\text{u}}_{\text{β}}}{\sin^{\text{-}1}\sqrt{\text{p}1}{\text{-sin}}^{\text{-1}}\sqrt{\text{p2}}}\right]}^{2}$$

According to the preliminary experiment conducted in the early stage of this study, the SC rate in the SC clinic group was 45%, and in the WeChat mini-program group it was 25%. Therefore, we set p1=0.45, p2=0.25, α=0.05, β=0.10, and calculated a required sample size of n=115 for each group. Hence, in this study, both the SC clinic group and the WeChat mini-program group needed 115 cases each. Considering a 20% loss to follow-up rate, the sample size for both groups was set at ≥138 cases.

### Statistical analysis

Statistical analyses were performed using SPSS 23.0. Categorical data were expressed as frequencies and percentages, and analyzed using the chi-squared test. Normally distributed continuous variables were expressed as means and standard deviations, while non-normally distributed continuous variables were expressed as medians and interquartile ranges. For normally distributed continuous variables, the t-test was employed, while the Mann-Whitney U test was used for non-normally distributed continuous variables. Logistic regression analysis was used to identify the factors influencing SC success at 3 months by evaluating the ORs and 95% CIs. The significance level was set at α_in_=0.05 and α_out_=0.10. A p<0.05 was considered statistically significant.

## RESULTS

### Demographic data

**Figure 3:**
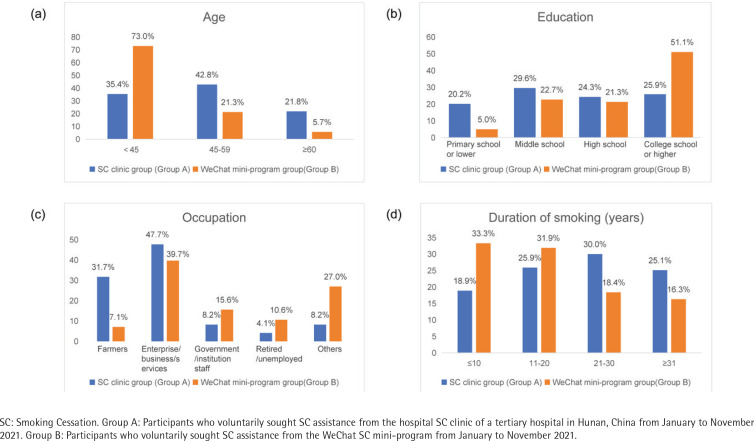
(a), (b), (c), (d) Comparison of age, education, occupation and duration of smoking between Groups A and Group B

Among the 395 participants recruited for the study, 11 individuals with incomplete data were excluded; thus, a final sample of 384 participants was included, including 243 in Group A (participants who voluntarily sought SC assistance from the hospital SC clinic) and 141 in Group B (participants who voluntarily sought SC assistance from the WeChat SC mini-program). The median patient age was 45 years. The majority of the participants in both groups were male (95.9% vs 95.7%), with median ages of 51 and 35 years, respectively. Most participants had a BMI of 18.5–23.9 (kg/m^2^) (56.0% vs 46.8%) (p=0.252). Two groups showed no statistically significant differences in BMI and gender (p>0.05). However, the groups did show statistically significant differences in terms of age, education level, and occupation (all p<0.05). In Group A, the majority of participants were aged 45–59 years (42.8%), whereas in Group B, the majority were aged <45 years (73.0%), with only 5.7% of the participants aged ≥60 years (p=0.002). The distribution of education level in Group A was relatively balanced, with the proportions of participants categorized as primary school or lower, middle school, high school, and college or higher, being 20.2%, 29.6%, 24.3%, and 25.9%, respectively. In Group B, the majority of participants had a college or higher (51.1%) education level, whereas only 5.0% had a primary school or lower (p<0.001). In terms of occupation, participants in both Group A and B were engaged in the enterprise/business/services industries, farmers, government/institutions staff, and other occupations (such as freelancers) (47.7%, 31.7%, 8.2%, 8.2% vs 39.7%, 7.1%, 15.6%, 27.0%) (p<0.001) (Table 1 and Figure 3).

**Table 1:** Comparison of demographic characteristics between the SC clinic group (Group A) and the WeChat mini-program group (Group B) participants (N=384)

*Characteristics*	*Total (N=384)*	*SC clinic group (Group A) (N=243)*	*WeChat mini-program group (Group B) (N=141)*	*χ^2^/Z*	*p*
	*n (%)*	*n (%)*	*n (%)*		
**Gender**				0.004	0.947
Female	16 (4.2)	10 (4.1)	6 (4.3)		
Male	368 (95.8)	233 (95.9)	135 (95.7)		
**Age** (years), mean ± SD		48.8 ± 13.4	36.6 ± 12.8	-3.076	0.002
<45	189 (49.2)	86 (35.4)	103 (73.0)		
45–59	134 (34.9)	104 (42.8)	30 (21.3)		
≥60	61 (15.9)	53 (21.8)	8 (5.7)		
**Education level**				32.106	0.000
Primary school or lower	56 (14.6)	49 (20.2)	7 (5.0)		
Middle school	104 (27.1)	72 (29.6)	32 (22.7)		
High school	89 (23.2)	59 (24.3)	30 (21.3)		
College school or higher	135 (35.2)	63 (25.9)	72 (51.1)		
**Occupation**				56.072	0.000
Farmers	87 (22.7)	77 (31.7)	10 (7.1)		
Enterprise/business/services	172 (44.8)	116 (47.7)	56 (39.7)		
Government/institution staff	42 (10.9)	20 (8.2)	22 (15.6)		
Retired/unemployed	25 (6.5)	10 (4.1)	15 (10.6)		
Other	58 (15.1)	20 (8.2)	38 (27.0)		
**BMI** (kg/m^2^)				-1.146	0.252
<18.5	16 (4.2)	8 (3.3)	8 (5.7)		
18.5–23.9	202 (52.6)	136 (56.0)	66 (46.8)		
24–26.9	103 (26.8)	65 (26.7)	38 (27.0)		
27–29.9	45 (11.7)	24 (9.9)	21 (14.9)		
≥30	18 (4.7)	10 (4.1)	8 (5.7)		

Group A: Participants who voluntarily sought SC assistance from the hospital SC clinic of a tertiary hospital in Hunan, China, from January to November 2021. Group B: Participants who voluntarily sought SC assistance from the WeChat SC mini-program from January to November 2021. SC: smoking cessation. BMI: body mass index.

### Smoking characteristics

The average smoking duration in Group A and Group B was 25.1 ± 12.8 years and 17.5 ± 11.4 years, respectively, while the average number of cigarettes smoked per day was 25.1 ± 13.3 and 20.2 ± 10.8, respectively. In Group A, participants who smoked ≥21 cigarettes per day and had a smoking duration of >20 years accounted for the largest proportions (42.8% and 55.1%, respectively). In Group B, the majority of participants smoked 11–20 cigarettes per day (48.2%) and had a smoking duration of ≤20 years (65.2%). The difference in smoking duration between the two groups was statistically significant (p=0.028), but the two groups showed no statistically significant difference in daily cigarette consumption (p=0.847).

Most participants had previously tried to QS (Group A: 51.0%; Group B: 67.4%). A statistically significant difference was observed between the two groups in terms of attempted SC (p=0.002). The median FTND score was 5. The average FTND scores for Groups A and B were 5.1 ± 2.9 and 5.0 ± 2.5, respectively; the FTND scores did not differ significantly between the two groups (p=0.895).

### Difficulty and reasons for quitting smoking

The SC difficulty scores of 0–60, 61–80, and 81–100 accounted for 40.7%, 36.6%, and 22.6% of the participants in Group A, respectively, and for 35.5%, 32.6%, and 31.9% of those in Group B, respectively. The SC difficulty score differed significantly between the two groups (p<0.001). In both groups, the reasons for SC were participants’ concerns about their health and that of their families (66.3% vs 61.0%), illness (27.2% vs 19.1%), being affected by the surrounding environment (2.5% vs 8.5%), and other reasons (such as preparing for pregnancy) (4.1% vs 11.3%). The reasons for SC also differed significantly between the two groups (p=0.001) (Table 2).

**Table 2:** Clinical characteristics of participants in the SC clinic group and the WeChat mini-program group (N=384)

*Characteristics*	*Total (N=384)*	*SC clinic group (Group A) (N=243)*	*WeChat mini-program group (Group B) (N=141)*	*χ^2^/Z*	*p*
	*n (%)*	*n (%)*	*n (%)*		
**Mean duration of smoking** (years), mean ± SD		25.1 ± 12.8	17.5 ± 11.4	-2.192	0.028
**Duration of smoking** (years)					
≤10	93 (24.2)	46 (18.9)	47 (33.3)		
11–20	108 (28.1)	63 (25.9)	45 (31.9)		
21–30	99 (25.8)	73 (30.0)	26 (18.4)		
≥31	84 (21.9)	61 (25.1)	23 (16.3)		
**Mean daily cigarettes**, mean ± SD		25.1 ± 13.3	20.2 ± 10.8	-0.193	0.847
**Cigarettes per day**					
≤10	78 (20.3)	43 (17.7)	35 (24.8)		
11–20	164 (42.7)	96 (39.5)	68 (48.2)		
≥21	142 (37.0)	104 (42.8)	38 (27.0)		
**SC attempts**				9.730	0.002
No	165 (43.0)	119 (49.0)	46 (32.6)		
Yes	219 (57.0)	124 (51.0)	95 (67.4)		
**Mean Fagerström score**, mean ± SD		5.1 ± 2.9	5.0 ± 2.5	-0.132	0.895
**Fagerström score**					
≤3	113 (29.4)	72 (29.6)	41 (29.1)		
4–6	144 (37.5)	84 (34.6)	60 (42.6)		
≥7	127 (33.1)	87 (35.8)	40 (28.4)		
**SC difficulty score^a^**				-3.909	0.000
0–60	149 (38.8)	99 (40.7)	50 (35.5)		
61–80	135 (35.2)	89 (36.6)	46 (32.6)		
81–100	100 (26.0)	55 (22.6)	45 (31.9)		
**Reasons and motivation for quitting smoking**				16.589	0.001
Concerned about own and their family’s health	247 (64.3)	161 (66.3)	86 (61.0)		
Their own illness	93 (24.2)	66 (27.2)	27 (19.1)		
Affected by the surrounding environment	18 (4.7)	6 (2.5)	12 (8.5)		
Other	26 (6.8)	10 (4.1)	16 (11.3)		

Group A: Participants who voluntarily sought SC assistance from the hospital SC clinic of a tertiary hospital in Hunan, China, from January to November 2021. Group B: Participants who voluntarily sought SC assistance from the WeChat SC mini-program from January to November 2021. SC: smoking cessation.

a The SC difficulty score was determined through participants’ self-assessment of the difficulty of quitting, on a scale from 0 to 100. A higher score indicates a greater level of difficulty in quitting.

### SC rates and characteristics at 3 months

The cessation rates in Groups A and B were 42.4% (103/243) and 24.8% (35/141), respectively, at 1 month, and 35.4% (86/243) and 22.7% (32/141), respectively, at 3 months. The SC rate at 3 months was higher in Group A than in Group B. In terms of the reasons for SC, Group A included a higher proportion of participants who showed success due to concerns about their own and their family’s health (31.1% vs 19.8%) and personal illness (51.5% vs 29.6%). However, Group A included a lower proportion of participants whose success was influenced by the surrounding environment (0.0% vs 8.3%) and other reasons, such as preparing for pregnancy (20.0% vs 37.5%) (Table 3).

**Table 3:** Clinical characteristic of participants in the SC clinic group and the WeChat mini-program group in smoking cessation for three months

*Characteristics*	*Total (N=384) n (%)*	*SC clinic group (Group A) (N=243)*		*χ^2^*	*p*	*WeChat mini-program group (Group B) (N=141)*		*χ^2^*	*p*
		*Abstinence failed (N=157) n (%)*	*Abstinence succeeded (N=86) n (%)*			*Abstinence failed (N=109) n (%)*	*Abstinence succeeded (N=32) n (%)*		
**Duration of smoking**, (years)				2.204	0.528	2.786	0.432		
≤10	93 (24.2)	30 (65.2)	16 (34.8)			39 (83.0)	8 (17.0)		
11–20	108 (28.1)	45 (71.4)	18 (28.6)			35 (77.8)	10 (22.2)		
21–30	99 (25.8)	46 (63.0)	27 (37.0)			20 (76.9)	6 (23.1)		
≥31	84 (21.9)	36 (59.0)	25 (41.0)			15 (65.2)	8 (34.8)		
**Cigarettes per day**				2.117	0.353	1.206	0.552		
≤10	78 (20.3)	25 (58.1)	18 (41.9)			25 (71.4)	10 (28.6)		
11–20	164 (42.7)	67 (69.8)	29 (30.2)			55 (80.9)	13 (19.1)		
≥21	142 (37.0)	65 (62.5)	39 (37.5)			29 (76.3)	9 (23.7)		
**SC attempts**				0.001	0.975			0.448	0.503
No	165 (43.0)	77 (64.7)	42 (35.3)			34 (73.9)	12 (26.1)		
Yes	219 (57.0)	80 (64.5)	44 (35.5)			75 (78.9)	20 (21.1)		
**Fagerström score**				4.753	0.097	0.104	0.967		
≤3	113 (29.4)	43 (59.7)	29 (40.3)			31 (75.6)	10 (24.4)		
4–6	144 (37.5)	62 (73.8)	22 (26.2)			47 (78.3)	13 (21.7)		
≥7	127 (33.1)	52 (59.8)	35 (40.2)			31 (77.5)	9 (22.5)		
**SC difficulty score^a^**				9.169	0.010			5.948	0.052
0–60	149 (38.8)	53 (53.5)	46 (46.5)			34 (68.0)	16 (32.0)		
61–80	135 (35.2)	63 (70.8)	26 (29.2)			35 (76.1)	11 (23.9)		
81–100	100 (26.0)	41 (74.5)	14 (25.5)			40 (88.9)	5 (11.1)		
**Reasons and motivation for quitting smoking**					0.004*			4.648^b^	0.215
Concerned about own and their family’s health	247 (64.3)	111 (68.9)	50 (31.1)			69 (80.2)	17 (19.8)		
Their own illness	93(24.2)	32 (48.5)	34 (51.5)			19 (70.4)	8 (29.6)		
Affected by the surrounding environment	18(4.7)	6 (100.0)	0 (0.0)			11 (91.7)	1 (8.3)		
Other	26(6.8)	8 (80.0)	2 (20.0)			10 (62.5)	6 (37.5)		

a The SC difficulty score was determined through participants’ self-assessment of the difficulty of quitting, on a scale from 0 to 100. A higher score indicates a greater level of difficulty in quitting.

b Continuous correction value.

* Monte Carlo p. Group A: Participants who voluntarily sought SC assistance from the hospital SC clinic of a tertiary hospital in Hunan, China, from January to November 2021. Group B: Participants who voluntarily sought SC assistance from the WeChat SC mini-program from January to November 2021 . SC: smoking cessation.

### Determinants of successful SC at 3 months

We performed a multiple logistic regression analysis using the SC status at 3 months as the dependent variable and participant grouping, age, education level, occupation, smoking duration, SC attempts previously, SC difficulty score, and reasons for SC as independent variables. The two groups showed no statistically significant difference in the factors influencing SC success (p=0.692). However, other variables such as education level, occupation, SC difficulty score, and reasons for SC were found to be significant factors influencing SC.

In comparison with participants who had an education level of primary school or lower, those with a middle school education had a lower likelihood of successful SC (OR=0.38; 95% CI: 0.17–0.82, p=0.014). The likelihood of SC success for individuals in the enterprise/business/service sector was 3.53-fold higher than that for farmers (OR=3.53; 95% CI: 1.31–9.52, p=0.013). As the SC difficulty score increased from 0–60 to 61–80, and then to 81–100, the likelihood of SC success decreased. Higher SC difficulty scores were associated with a lower likelihood of successful SC (OR=0.38; 95% CI: 0.22–0.68, p=0.001; OR=0.27; 95% CI: 0.14–0.52, p<0.001).

Regarding the reasons for SC, participants who QS due to their illness showed a 2.23-fold higher likelihood of SC success than those who QS due to concerns about their own and their family’s health (OR=2.23; 95% CI: 1.26–3.93, p=0.006). Participants who QS for other reasons, such as preparing for pregnancy, had a 2.32-fold higher likelihood (OR=2.32; 95% CI: 0.86–6.31, p=0.098). Moreover, in comparison with participants who QS due to concerns about their own and their family’s health, those who QS due to the influence of the surrounding environment showed a lower likelihood of SC (OR=0.12; 95% CI: 0.01–1.06, p=0.057) (Table 4).

**Table 4:** Binary logistic regression analysis* of predictive factors for smoking cessation

*Variable*	*p*	*OR*	*95 % CI*
**Group**			
SC clinic group (Group A) (Ref.)		1	
WeChat mini-program group (Group B)	0.692	0.89	0.49–1.61
**Age** (years)			
<45 (Ref.)		1	
45–59	0.713	1.16	0.53–2.54
≥60	0.792	1.16	0.38–3.55
**Education level**			
Primary school or lower (Ref.)		1	
Middle school	0.014	0.38	0.17–0.82
High school	0.285	0.62	0.26–1.49
College school or higher	0.854	1.09	0.42–2.85
**Occupation**			
Farmers (Ref.)		1	
Enterprise/business/services	0.013	3.53	1.31–9.52
Government/institution staff	0.195	1.73	0.76–3.97
Retired/unemployed	0.330	0.56	0.18–1.79
Other	0.594	0.69	0.18–2.72
**Duration of smoking**, (years)			
≤10 (Ref.)		1	
11–20	0.756	0.89	0.42–1.87
21–30	0.688	1.20	0.49–2.91
≥31	0.433	1.55	0.52–4.60
**SC attempts**			
No (Ref.)		1	
Yes	0.717	1.10	0.66–1.82
**SC difficulty score^a^**			
0–60 (Ref.)		1	
61–80	0.001	0.38	0.22–0.68
81–100	0.000	0.27	0.14–0.52
**Reasons and motivation for quitting smoking**			
Concerned about own and their family’s health (Ref.)		1	
Their own illness	0.006	2.23	1.26–3.93
Affected by the surrounding environment	0.057	0.12	0.01–1.06
Other	0.098	2.32	0.86–6.31

SC: smoking cessation. Group A: Participants who voluntarily sought SC assistance from the hospital SC clinic of a tertiary hospital in Hunan, China from January to November 2021. Group B: Participants who voluntarily sought SC assistance from the WeChat SC mini-program from January to November 2021.

a The SC difficulty score was determined through participants’ self-assessment of the difficulty of quitting, on a scale from 0 to 100. A higher score indicates a greater level of difficulty in quitting.

* Based on a combination of univariate analysis results and professional judgment, the following variables were selected for binary logistic regression analysis: participant grouping, age, education level, occupation, smoking duration, SC attempts, SC difficulty score, and reasons for SC.

## DISCUSSION

### Comparison of the SC rate between Group A and B

Group A (participants who voluntarily sought SC assistance from the hospital SC clinic) and Group B (participants who voluntarily sought SC assistance from the WeChat SC mini-program) showed higher SC rates at 3 months than self-quitting rates without any intervention (35.4% and 22.7%, vs 4.0%)^18^. Cheung et al.^19^ reported that using WhatsApp or Facebook online social groups for SC resulted in higher cessation rates than those achieved with no intervention (38.0% vs 38.0% vs 24.0%; 26.0% vs 25.0% vs 15.0%), indicating that online SC interventions can effectively improve cessation rates.

In this study, the SC rate at 1 month in Group A was 42.4%, which was higher than that of the National Central Subsidy Smoking Cessation Clinic Project (34.1%)^20^. However, this rate was slightly lower than the follow-up at 1 month cessation rate of SC clinics in Chengdu (43.3%)^10^. The SC rate at 3 months in Group A (35.4%) was higher than that of the SC clinics in Hainan (22.8%)^14^ and that among male smokers in Beijing (25.5%)^21^. However, it was lower than the SC rate at 3 months reported for SC clinics in three locations in Tianjin (45.2%)^22^.

In this study, the SC rate at 3 months in Group B was 22.7%. This was similar to the follow-up cessation rates at 2 months (7-day point prevalence abstinence) of participants using SmartQuit 1.0 (23.0%) and SmartQuit 2.0 (21.0%)^23^. Marler et al.^24^ found that the Pivot mobile SC program and QuitGuide SC smartphone app had self-reported 7-day abstinence rates of 35.0% and 28.0%, respectively, after 12 weeks, which were higher than the rates obtained in this study. Liu et al.^25^ reported a 7-day cessation rate of 25.0% using the QuitGuide SC app, which was higher than the rate for non-users of SC apps. These findings indicate that both SC clinics and online SC interventions are effective in increasing cessation rates among smokers. Therefore, both represent important approaches that are worth promoting for SC.

### Analysis of factors influencing SC success among participants in Groups A and B

There was no difference observed in influencing SC success between Group A and Group B in this study. However, education level, occupation, SC difficulty score, and the reason for SC were identified as significant factors influencing SC success.

### Influence of education level and occupation on SC

This study shows that a higher proportion of young individuals chose mobile SC applications. They also had a higher educational level and were less engaged in physically demanding agricultural occupations. In contrast, those who chose SC clinics were predominantly middle-aged individuals with a high school education or lower, and mostly were farmers. These findings are consistent with the results of previous studies^8,10^. Xie et al.^26^ conducted a survey on the general characteristics of 841 smokers seeking SC services at clinics in Shanghai, and found that 92.0% of participants recruited were male; 76.4% were aged 35–55 years; 58.9% had an education level of high school or lower; and 47.8% were enterprise/business/service industry workers. Nash et al.^27^ investigated the characteristics of 141429 adult tobacco users who self-selected to join either a standalone web-based program (Web-Only) or an integrated phone/web program (Phone/Web) for SC, and the study showed that compared to participants in the Phone/Web program, those who chose the Web-Only program were younger (average age: 40.8 vs 45.3 years) and had a higher education level (above high school, 59.7% vs 44.2%). Pallejà-Millán et al.^8^ reported that young individuals were more likely to accept and utilize mobile SC applications, which reduced smoking rates as well as the occurrence of tobacco-related complications among young smokers. Zeng et al.^12^ reported that individuals with a higher educational level were significantly more likely to open a SmartQuit mobile application than individuals with a lower educational level (high school or lower), indicating that individuals with a higher educational level were more willing to accept and use new technologies. This study also showed that smokers who used SC mobile applications had a higher educational level, with university or higher accounting for 51.1% of smokers. In comparison with farmers, individuals engaged in light physically demanding work in the enterprise/business/service sector mainly work in urban areas, have better access to health knowledge, have a higher awareness of tobacco hazards, and are more concerned about health and seeking SC help.

### The higher the SC difficulty score, the lower the likelihood of SC success

In this study, participants with SC difficulty scores of 61–80 and 81–100 had a lower likelihood of successful SC than those with scores of 0–60, and the higher the SC difficulty score, the lower the likelihood of SC success. This finding was similar to the results reported by Xie et al.^28^. In another study, the sustained SC rate at 3 months was higher for smokers with difficulty scores of <8.0 (22.1%, 163/737) compared to those with scores ≥8.0 (17.8%, 142/797)^4^. This may be related to participants’ self-efficacy, since smokers with lower SC difficulty scores had more confidence and self-efficacy in QS, thus increasing the likelihood of SC success^29^.

Li et al.^13^ found that more than half of the smokers found quitting smoking difficult, indicating a lack of strong motivation and determination to QS and highlighting the need to enhance their motivation and willingness to QS. Smokers who had received persuasion from people around them to QS were more willing to attempt quitting, and those who had been exposed to anti-smoking campaigns and had a higher motivation were more likely to succeed in SC^30^. A positive correlation was observed among SC attempts, willingness, and motivation to QS^31^. These results highlight the benefits of increasing follow-up and counseling interventions for smokers with higher SC difficulty scores to improve SC rates.

### Reasons for quitting smoking vary in the likelihood of SC success

Regarding the reasons for quitting smoking in this study, participants in Group A were more likely to QS successfully due to their focus on their own and their family’s health and personal illness, while participants in Group B were more likely to have the intention to QS for other reasons, such as pregnancy preparation and environmental influences. Zhao et al.^9^ also indicated that the main reasons for smokers in the SC clinic to QS were their concern for their own and their families’ health (54.7%, 803/1467), personal illness (35.2%, 517/1467), environmental influences (8.5%, 124/1467), and other reasons (1.6%, 23/1467). This may be because some smokers in Group A obtained relevant information about the clinic through healthcare workers and hospital promotion, which made their intention to QS stronger than the participants in Group B and less susceptible to environmental influences^32^. These findings highlight the importance of promoting knowledge of tobacco hazards and increasing smokers’ determination to QS.

In this study, the likelihood of successful SC among participants who QS due to personal illness and other reasons (such as pregnancy) was 2.23- and 2.32-fold higher, respectively than the likelihood among those who QS because they were concerned for their own and their family’s health. The results of a study in Tianjin, indicated that SC effectiveness was slightly lower for those who QS because of their concern for their own and their family’s health compared to those who QS due to personal illness (OR=1.96; 95% CI: 1.43–2.69)^33^, this result is consistent with the findings of the present study. Participants who had a personal illness or were preparing for pregnancy were more eager to QS as a means to improve their own and their family’s health^34^. In this study, the likelihood of SC among participants influenced by their environment was lower. In a study by Wu et al.^21^, male smokers were more likely to fail to QS when influenced by other smokers in their surroundings, whereas male smokers with tobacco-related chronic diseases were more likely to succeed in quitting smoking. This finding suggests a possible association between SC success and the influence of companions who are smokers. Van et al.^35^ reported a significant correlation between smokers’ social environment and successful SC. Workplace SC programs can assist smokers with lower QS motivation levels to QS and prevent relapses^36^. Therefore, tobacco control campaigns in public places and the creation of supportive SC environments are crucial for people who are ready to QS.

### Limitations

This study has some limitations. The SC rates of the participants in both groups were obtained through self-reported results without biochemical verification, which may have introduced a bias into the results. This study was conducted in Hunan, China, with a relatively small fraction of female participants. This could potentially introduce residual confounding, result in the underrepresentation of women, limit the generalizability of findings to other countries, and present additional limitations. This study compared the SC rates of participants who chose the SC clinic at our hospital with those who used the WeChat SC mini-program developed by our team. The effectiveness of the WeChat SC mini-program could be further evaluated and improved by collaborating with other hospitals and comparing the findings with other SC platforms in a multicenter study with a larger sample size in the future.

## CONCLUSIONS

Both SC clinics and SC mini-programs can effectively help smokers QS and improve SC rates. Participants using the SC mini-program tended to be younger, had a higher education level (college school or higher), worked in urban areas, had a smoking history of mostly ≤20 years, and showed a higher proportion of previous attempts to QS. They were also more likely to QS due to environmental influences and other reasons. However, individuals with middle school education level and farmers were less likely to succeed in quitting smoking than those with primary school or lower education, and individuals employed in the enterprise/business/services industries. A higher difficulty score in SC was an independent risk factor. Participants who were attempting to QS due to personal illness and other reasons (such as pregnancy) had a higher likelihood of successful SC than those who were attempting to QS primarily due to their concern for their own and their family’s health. Individuals influenced by their environment showed a lower likelihood of successful SC. These findings highlight the importance of promoting and encouraging the use of both SC mini-programs and SC clinics to improve SC rates through effective advertising and push notifications.

## Data Availability

The data supporting this research are available from the authors on reasonable request.
